# Everybody's Page

**Published:** 1892-03-19

**Authors:** 


### Everybody's Page.

The Association for Improving the Condition of the Poor in New York has taken to erecting baths, in which the charge for a bath, including a new cake of soap, and the use of a large towel, is only five cents. With a view, however, we suppose, to encouraging habits of cleanliness this modest fee is remitted to those who cannot afford to pay. It would be interesting to know how many applicants for admission allege the necessary qualification for free admission.

A five per cent. solution of carbolic acid in water is advocated by some French dentists as a safer and more reliable local anæsthetic in dentistry than cocaine, being equally efficacious and less dangerous. The mode of operation is to inject four or five drops under the gum on each side of the tooth to be extracted, the action being more especially satisfactory where inflammation exists round the root. It is also asserted, that with moderate care, owing to the small quantity of carbolic acid used, there is no danger of constitutional symptoms arising.

The appointment of a lady as a medical practitioner in a town in Bosnia is a triumph over the fanaticism of the Turkish authorities who by nature averse to reforms are especially opposed to the introduction of Western ideas and ways of living which are so repugnant to the orthodoxy of their elders. Owing to the fact that Mahometan women have been deprived of the services of medical men, who are forbidden access to the harem, however grave their illnesses may have been, the spread of disease has been practically unchecked. If the Government in the occupied provinces carry out their intention of appointing ladies as doctors in the Mahometan settlements, the days of female sooth sayers and quacks will, we trust, soon be numbered.

A very sensible regulation has recently been enforced by the Minister of Public Instruction in France, to the effect that in all schools and colleges filtered water must be used not only for drinking purposes but for washing salad and glasses and tumblers. It is quite as important that our salads should be washed with pure water as that the water we drink should be freed from all taint, though this point is frequently lost sight of by the householder.

An American doctor reports a case of a woman, seventy-one years of age, who sheds her bones—not a pleasant occupation either for herself or her friends. The doctor reports her as being "always cheerful, a very interesting fact as showing the power of the mind to adapt itself to extraordinary circumstances." We quite agree with the doctor that a mind which can adapt itself cheerfully to the shedding of bones must be capable of great things. So far the lady has only made her mark in the history of her country by this remarkable feat, and by presenting her bones to friends as souvenirs!

Depilatory powders are, no doubt, in great demand by certain unfortunate people to whom nature has been too bountiful in the gift of that ornament which, if it grows in profusion in the right place is the glory of the fair sex. But such powders are not to be used with impunity, any more than the other powders and washes which are at the same time the burning hope and bitter despair of the budding youth who aspires to a military moustache, but only acquires a few blisters on his lip in return for his enthusiasm. Sulphohydrate of sodium when used as a depilatory is likely, if not quite fresh, to produce scars, and those who wish to indulge in such a luxury will probably find sulphide of barium safer.

